# Similar early mortality risk after cemented compared with cementless total hip arthroplasty for primary osteoarthritis: data from 188,606 surgeries in the Nordic Arthroplasty Register Association database

**DOI:** 10.1080/17453674.2020.1842003

**Published:** 2020-11-04

**Authors:** Alma B Pedersen, Aurélie Mailhac, Anne Garland, Søren Overgaard, Ove Furnes, Stein Atle Lie, Anne Marie Fenstad, Cecilia Rogmark, Johan Kärrholm, Ola Rolfson, Jaason Haapakoski, Antti Eskelinen, Keijo T Mäkelä, Nils P Hailer

**Affiliations:** a Department of Clinical Epidemiology, Aarhus University Hospital, Aarhus, Denmark;; b Department of Surgical Sciences/Orthopedics, Uppsala University, Uppsala, Sweden;; c Department of Orthopedic Surgery and Traumatology, Odense University Hospital, Department of Clinical Research, University of Southern Denmark, Odense, Denmark, and the Danish Hip Arthroplasty Register;; d The Norwegian Arthroplasty Register, Department of Orthopedic Surgery, Haukeland University Hospital, Bergen, Norway;; e Department of Clinical Medicine, University of Bergen, Norway;; f Department of Clinical Dentistry, University of Bergen, Bergen, Norway;; g Department of Orthopedics, Lund University, Skåne University Hospital, Malmö, Sweden;; h The Swedish Hip Arthroplasty Registry, Registercentrum Västra Götaland, Gothenburg, Sweden;; i Department of Orthopaedics, Institute of Clinical Sciences, Sahlgrenska Academy, University of Gothenburg, Sweden;; j Finnish Arthroplasty Register, National Institute for Health and Welfare, Helsinki, Finland;; k Coxa Hospital for Joint Replacement, and Faculty of Medicine and Health Technologies, University of Tampere, Tampere, Finland;; l Department of Orthopedics and Traumatology, Turku University Hospital, Turku, Finland

## Abstract

Background and purpose — Current literature indicates no difference in 90-day mortality after cemented compared with cementless total hip arthroplasty (THA). However, previous studies are hampered by potential selection bias and suboptimal adjustment for comorbidity confounding. Therefore, we examined the comorbidity-adjusted mortality up to 90 days after cemented compared with cementless THA performed due to osteoarthritis.

Patients and methods — Using the Nordic Arthroplasty Register Association database, 2005–2013, we included 108,572 cemented and 80,034 cementless THA due to osteoarthritis. We calculated the Charlson comorbidity index of each patient based on data from national patient registers. The Kaplan–Meier method was used to estimate unadjusted all-cause mortality. Cox regression was used to estimate hazard ratios (HR) with 95% confidence intervals (CI) for 14, 30-, and 90-day mortality comparing cemented with cementless THA, adjusting for age, sex, comorbidity, nation, and year of surgery.

Results — Cumulative all-cause mortality within 90 days was 0.41% (CI 0.37–0.46) after cemented and 0.26% (CI 0.22–0.30) after cementless THA. The adjusted HR for cemented vs. cementless fixation was 0.97 (CI 0.79–1.2), and similar risk estimates were obtained for mortality within 14 (adjusted HR 0.91 [CI 0.64–1.3]) and 30 days (adjusted HR 0.94 [CI 0.71–1.3]). We found no clinically relevant differences in mortality between cemented and cementless THA in analyses stratified by age, sex, Charlson comorbidity index, or year of surgery.

Interpretation — After adjustment for comorbidity as an important confounder, we observed similar early mortality between the 2 fixation techniques.

The question of whether cemented fixation of total hip arthroplasty (THA) increases early postoperative mortality, potentially by inducing bone cement implantation syndrome (BCIS) or thromboembolism, is fiercely debated (Olsen et al. 2014). The 90-day mortality after THA is reported to be 0.7% in a systematic review based on 32 observational studies including 1,129,330 patients (Berstock et al. 2014). The 3 most frequent causes of death within 90 days after THA are myocardial infarction, thromboembolism, and pneumonia (Pedersen et al. 2011). Since death is a rare complication after THA, only large register studies will have the statistical power to address the question of whether cemented THA fixation indeed confers an increased risk of early mortality.

Analyses based on Norwegian, Finnish, or Swedish register data indicate similar 90-day mortality after cemented compared with cementless THA, but only a few studies were designed to adequately address comorbidity confounding using different methods (Garland et al. 2017, Dale et al. 2019, Ekman et al. 2019). The Norwegian study on 79,557 patients had access to ASA class, based on physicians’ preoperative assessment of overall health (Dale et al. 2019), whereas the Finnish study on 62,221 patients had information on comorbid conditions based on a hospital discharge register (Ekman et al. 2019). A Swedish study on 178,784 patients compared mortality after cemented and cementless THA adjusting for the Charlson comorbidity index (CCI), but the cohort exposed to cementless fixation was only about a 10th the size of the cohort exposed to cemented THA (Garland et al. 2017). That study is also hampered by selection bias since the practice in Sweden is to select younger and fitter patients for cementless fixation. The Nordic Arthroplasty Register Association (NARA) was established to fuse 4 well-established Nordic arthroplasty databases into a larger framework (Havelin et al. 2009). The choice of fixation techniques varies considerably among the participating countries, with a predominance of cemented fixation in Sweden and Norway and a much larger proportion of cementless fixation in Denmark and Finland (Mäkelä et al. 2014b). Thus, the motivation for performing our study was the possibility to cope with the selection bias by analysing patients from all Nordic countries together and to improve the adjustment for comorbidity confounding compared with previous studies. In addition, we examine whether age, sex, and calendar year are effect modifiers in the association between fixation and mortality, which was not examined in previous studies.

We thus designed an international register-based cohort study aimed at comparing mortality within 0–14, 0–30, and 0–90 days after cemented and cementless THA performed due to osteoarthritis with adjustment for comorbidities at the time of surgery. 

## Patients and methods

### Study design and sources of data

The study was designed as a population-based cohort study on primary THA procedures performed in Denmark, Finland, Norway, and Sweden during 2005–2013, and the cohort was obtained from the combined NARA database. The database includes a common set of variables collected in all participating countries. Due to personal identification numbers used in all Nordic countries it is possible to further link national arthroplasty register data to data from national patient registries in each country, and then to anonymize and transfer data to a common NARA database.

### Study population

Using the NARA database, we included all primary cemented and cementless THAs performed due to osteoarthritis during the period 2005 to 2013. Only 1st unilateral THAs were included. Some of the exclusion criteria were already applied on the national level before transferring data to NARA, whereas other exclusion criteria were applied in the NARA dataset. The hybrid THA were excluded from the study population. Thus, the final study population included 188,606 THA patients (Table 1). The index date was defined as the date of THA.

**Table 1.** Flow diagram ^a^

		Finland All OA, first- time surgery	Denmark All OA 2005–2013	Norway All OA 2005–2013	Sweden All OA 2005–2013
No. received from each country	76,066	58,087	49,208	100,383	
Keep 2005–2013	51,469				
Keep only cemented/cementless implants (hybrids are excluded)	42,452	48,015	34,721	86,193	
Keep non-simultaneous	40,618	47,361	34,639	85,215	
Keep first surgery		41,474	30,907	75,608	
Remove negative time to death	40,617				
Final study population	40,617	41,474	30,907	75,608	

**^a^** Some of the exclusion criteria were already applied on the national level before transferring data to NARA, whereas other exclusion criteria were applied in the NARA dataset.

### Exposure

The exposure was THA fixation technique, including cemented and cementless THA. Hybrid THAs were not included in our study since the aim was not to examine the association between exposure to hybrids and mortality.

### Outcome

The outcome was time to death within up to 90 days of the index date. Information on death was available from the central statistics facilities in Denmark (Schmidt et al. 2014), Finland, Norway, and Sweden and was linked to each patient in the national registers before transferring the data to NARA database.

### Potential confounders

The following variables were included from the NARA database as potential confounders: age (≤ 59, 60–69, 70–79, and ≥ 80 years), sex, nation (Denmark, Norway, Finland, and Sweden), and year of THA (2005–2013).

The level of comorbidity in each individual participant was assessed during the 2 years preceding the index date. The information on comorbidity was collected from the National Discharge Registries of Finland (Sund 2012), Denmark (Schmidt et al. 2015), Sweden (Ludvigsson et al. 2011), and Norway (Bakken et al. 2020) before transferring to the NARA database in anonymized form. We calculated the CCI in the adaptation suggested by Quan et al. (2011) (Charlson et al. 1987) and categorized comorbidities into 4 CCI scores: low (score 0: no known comorbidities), mild (score 1), moderate (score 2), and severe (score 3 and more). The look-back period for CCI calculation was 2 years prior to the index date and during the hospitalization for the index THA for the 19 comorbidity groups included in CCI and comorbidities that are not part of the CCI were not considered.

### Statistics

We tabulated characteristics of the study population by fixation technique. Numbers of patients and proportions in each category are presented. Means were used to describe age. Categorical data were summarized in cross-tables by fixation technique.

We used the Kaplan–Meier method to estimate unadjusted cumulative survival probability by fixation technique. We fitted Cox regression models to estimate hazard ratios (HR) with 95% confidence intervals (CI). Overall HRs were adjusted for age (as continuous variable), sex, CCI score, nation, and year of THA surgery, and the inclusion of these covariates was based on the assessment of relevance and non-interference using directed acyclic graphs. Furthermore, we studied the association between fixation and mortality stratifying on age, sex, CCI score, and year of index THA surgery. We tested for interaction between age and fixation, as well as sex, nation and year of surgery and fixation. P-values less than 0.05 were considered statistically significant.

We used the non-parametric Aalen additive regression model as a sensitivity analysis in order to assess graphically whether there were time-dependent effects of THA fixation on an additive scale, as opposed to the semi-parametric multiplicative Cox regression model.

Follow-up started on the index date and ended on the day of death, or December 31, 2013, whichever came first. We used SAS version 9.4 (SAS Institute, Cary, NC, USA) to perform data management and analyses. The study follows the RECORD guidelines (Benchimol et al. 2015).

### Ethics, funding, and potential conflicts of interest

Ethical approval was granted by the appointed authority in each participating country: the Ethics Boards of Lund (LU20-02) and Gothenburg (360/13 and T453/14) in Sweden, the Finnish National Institute of Health and Welfare (Dnro THL/1743/5.05.00/2014), the Norwegian Data Inspectorate (ref 24.1.2017: 16/01622-3/CDG and Ethical approval 2015/880/REK Vest), and the Danish Data protection agency (1-16-02-54-17). Data sharing is not possible. The study was supported by a grant from Aarhus University Research Foundation and by the Swedish Research Council (VR 2018-02612). The funders had no role in the design and conduct of the study; collection, management, analysis, and interpretation of the data; preparation, review, or approval of the manuscript; and decision to submit the manuscript for publication.

All authors have completed the ICMJE uniform disclosure form at http://www.icmje.org/conflicts-of-interest/ (available on request from the corresponding author) and declare that (1) no authors have received support from any company for the submitted work. The Department of Clinical Epidemiology at Aarhus University Hospital is, however, involved in studies with funding from various companies as research grants to (and administered by) Aarhus University. None of these studies have any relation to the present study; (2) no authors have personal financial relationships with any company that might have an interest in the submitted work in the previous 3 years; (3) no authors have non-financial interests that may be related to the submitted work. However, AE reports grants from DePuy Synthes, grants from Zimmer Biomet, personal fees from Zimmer Biomet, outside the submitted work. SO reports grants from Zimmer Biomet, outside the submitted work. NH reports grants and lecturer’s fees by Waldemar Link GmbH & Co. KG, Hamburg, and Heraeus GmbH, Wehrheim, Germany, outside the submitted work.

## Results

### Characteristics of the study population

Among the 188,606 included THA procedures, 58% were performed using cemented and 42% using cementless fixation (Table 2). The proportion of females in the entire study population was 58%, mean age at index surgery was 69 years (SD 9.7), and the majority of patients (83%) presented without comorbidities classified according to the modified CCI. The two fixation cohorts differed with respect to some baseline characteristics (Table 2). The proportion of females was higher in the cohort receiving cemented fixation, the proportion of patients younger than 60 years was considerably higher in the cohort operated with cementless fixation, and the proportion of patients with varying degrees of comorbidity was slightly higher among those receiving cemented fixation.

**Table 2.** Baseline characteristics of patients with cemented and cementless total hip arthroplasty (THA) in the Nordic Arthroplasty Registry Association database by country, 2005–2013. Values are count (%)

Fixation:	All patients		Denmark		Norway		Sweden		Finland	
	Cemented	Cementless	Cemented	Cementless	Cemented	Cementless	Cemented	Cementless	Cemented	Cementless
	n = 108,572	n = 80,034	n = 9,954	n = 31,520	n = 22,772	n = 8,135	n = 64,481	n = 11,127	n = 11,365	n = 29,252
Sex										
Male	39,655 (37)	38,789 (49)	3,446 (35)	15,452 (49)	6,957 (31)	3,091 (38)	25,601 (40)	6,279 (56)	3,651 (32)	13,967 (48)
Female	68,917 (63)	41,245 (51)	6,508 (65)	16,068 (51)	15,815 (69)	5,044 (62)	38,880 (60)	4,848 (44)	7,714 (68)	15,285 (52)
Age at primary THA										
≤ 59	5,720 (5.3)	22,525 (28)	215 (2.2)	7,017 (22)	1,132 (5.0)	2,188 (27)	4,186 (6.5)	6,147 (55)	187 (1.6)	7,173 (25)
60–69	29,760 (27)	33,165 (41)	1,305 (13)	13,981 (44)	5,843 (26)	3,096 (38)	21,050 (33)	4,048 (36)	1,562 (14)	12,040 (41)
70–79	49,377 (46)	20,033 (25)	5,418 (54)	8,659 (28)	10,326 (45)	2,076 (26)	27,250 (42)	798 (7.2)	6,383 (56)	8,500 (29)
≥ 80	23,715 (22)	4,311 (5.4)	3,016 (30)	1,863 (5.9)	5,471 (24)	775 (9.5)	11,995 (19)	134 (1.2)	3,233 (28)	1,539 (5.3)
Charlson comorbidity index										
0, none	89,105 (82)	66,939 (84)	7,119 (72)	25,153 (80)	19,646 (86)	7,028 (86)	53,210 (82)	10,085 (91)	9,130 (80)	24,673 (84)
1, mild	10,268 (9.5)	6,915 (8.6)	1,370 (14)	3,216 (10)	1,828 (8.0)	698 (8.6)	5,905 (9.2)	621 (5.6)	1,165 (10)	2,380 (8.1)
2, moderate	6,499 (6.0)	4,388 (5.5)	961 (9.7)	2,182 (6.9)	852 (3.7)	296 (3.6)	3,892 (6.0)	316 (2.8)	794 (7.0)	1,594 (5.4)
3, severe	2,700 (2.5)	1,792 (2.2)	504 (5.1)	969 (3.1)	446 (2.0)	113 (1.4)	1,474 (2.3)	105 (0.9)	276 (2.4)	605 (2.1)
Year of primary THA										
2005	17,385 (16)	5,446 (6.8)	1,795 (18)	2,186 (6.9)	3,663 (16)	595 (7.3)	9,415 (15)	767 (6.9)	2,512 (22)	1,898 (6.5)
2006	15,654 (14)	6,278 (7.8)	1,610 (16)	2,456 (7.8)	3,218 (14)	578 (7.1)	8,719 (14)	997 (9.0)	2,107 (19)	2,247 (7.7)
2007	14,396 (13)	7,145 (8.9)	1,438 (14)	2,901 (9.2)	3,351 (15)	461 (5.7)	8,031 (13)	1,250 (11)	1,576 (14)	2,533 (8.7)
2008	13,078 (12)	8,080 (10)	1,041 (10)	3,036 (9.6)	3,021 (13)	663 (8.1)	7,631 (12)	1,368 (12)	1,385 (12)	3,013 (10)
2009	12,537 (12)	9,904 (12)	866 (8.7)	4,401 (14)	2,626 (12)	860 (11)	8,004 (12)	1,502 (14)	1,041 (9.2)	3,141 (11)
2010	11,587 (11)	10,681 (13)	938 (9.4)	4,074 (13)	2,001 (8.8)	1,169 (14)	7,843 (12)	1,671 (15)	805 (7.1)	3,767 (13)
2011	10,831 (10)	11,226 (14)	820 (8.2)	4,120 (13)	1,803 (7.9)	1,196 (15)	7,476 (12)	1,777 (16)	732 (6.4)	4,133 (14)
2012	10,221 (9.4)	11,576 (15)	727 (7.3)	4,134 (13)	1,522 (6.7)	1,303 (16)	7,362 (11)	1,795 (16)	610 (5.4)	4,344 (15)
2013	2,883 (2.7)	9,698 (12)	719 (7.2)	4,212 (13)	1,567 (6.9)	1,310 (16)			597 (5.3)	4,176 (14)

Aalen additive plot with cumulative coefficients (y-axis) with 95% confidence interval comparing cemented with cementless total hip arthroplasties within 90 days of surgery.

**Figure:**
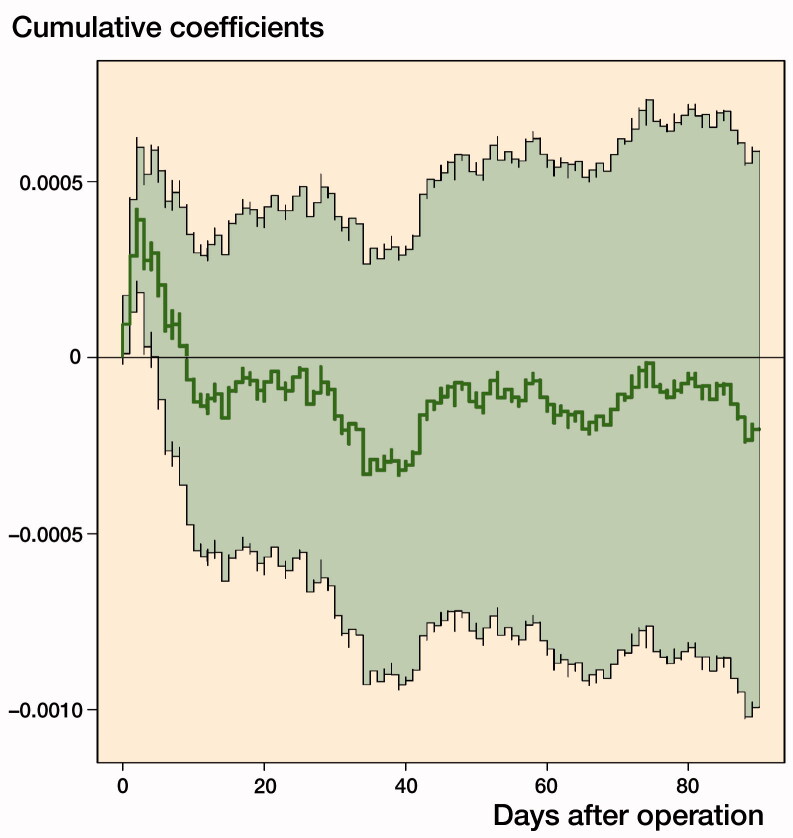


The majority of all cemented procedures were performed in Sweden (59%), whereas most cementless procedures were registered in Finland and Denmark (36% and 39%). During the period 2005–2008, cemented fixation was more frequently used than cementless fixation in all 4 countries, whereas in the period 2009–2013 we observed the opposite pattern.

### Risk of 90-day mortality

During the 1st 90 days of follow-up, the cumulative mortality after cemented and cementless THA procedures was estimated at 0.41% (CI 0.37–0.46) and 0.26% (CI 0.22–0.30), respectively (with 449 deaths after cemented and 208 deaths after cementless THA procedures). This was equivalent to an unadjusted HR of 1.6 (CI 1.4–1.9) for cemented compared with cementless fixation, but after adjustment for age, sex, CCI score, nation, and year of index THA, an HR of 0.97 (CI 0.79–1.2) was attained for cemented compared with cementless fixation.

We then analyzed the adjusted risk of 90-day mortality in 4 separate age groups and found similar 90-day mortality between cemented and cementless fixation in the age groups 60–69, 70–79, and > 80 years of age. However, cemented fixation conferred an increased risk of death among patients below the age of 59, with an adjusted HR of 3.5 (CI 1.4–8.9; Table 3), but the absolute risk difference was 0.09 (CI –0.04 to 0.21). We tested for interaction between age, sex, nation, and year of surgery and fixation within 90 days and all p-values were > 0.05.

**Table 3.** Crude and adjusted hazard ratios (HR) with 95% confidence interval (CI) for 90-day mortality comparing cemented with cementless total hip arthroplasties (THA) ^a^

Fixation	Number of deaths	Number at risk	Crude HR (95% CI)	Adjusted HR (95% CI)
Among all				
Cementless	208	80,034	Reference	Reference
Cemented	449	108,572	1.6 (1.4–1.9)	0.97 (0.79–1.2)
Stratified by age				
≤ 59				
Cementless	24	22,525	Reference	Reference
Cemented	11	5,720	1.8 (0.88–3.7)	3.5 (1.4–8.9)
60–69				
Cementless	51	33,165	Reference	Reference
Cemented	37	29,760	0.81 (0.53–1.2)	1.6 (0.63–2.1)
70–79				
Cementless	78	20,033	Reference	Reference
Cemented	164	49,377	0.85 (0.65–1.1)	1.1 (0.76–1.5)
≥ 80				
Cementless	55	4,311	Reference	Reference
Cemented	237	23,715	0.78 (0.58–1.1)	1.1 (0.77–1.5)
Stratified by gender				
Male				
Cementless	136	38,789	Reference	Reference
Cemented	212	39,655	1.5 (1.2–1.9)	0.81 (0.61–1.1)
Female				
Cementless	72	41,245	Reference	Reference
Cemented	237	68,917	2.0 (1.5–2.6)	1.2 (0.88–1.7)
Stratified by Charlson comorbidity index				
0, no comorbidity				
Cementless	128	66,939	Reference	Reference
Cemented	270	89,105	1.6 (1.3–2.0)	0.83 (0.63–1.1)
1, mild comorbidity				
Cementless	41	6,915	Reference	Reference
Cemented	69	10,268	1.1 (0.77–1.7)	1.1 (0.67–1.8)
2, moderate comorbidity				
Cementless	16	4,388	Reference	Reference
Cemented	46	6,499	1.9 (1.1–3.4)	1.2 (0.60–2.6)
3, severe comorbidity				
Cementless	23	1,792	Reference	Reference
Cemented	64	2,700	1.9 (1.2–3.0)	1.3 (0.72–2.3)
Stratified by year of primary operation				
2005–2008				
Cementless	75	26,949	Reference	Reference
Cemented	255	60,513	1.5 (1.2–2.0)	0.83 (0.61–1.1)
2009–2013				
Cementless	133	53,085	Reference	Reference
Cemented	194	48,059	1.6 (1.3–2.0)	1.2 (0.92–1.6)

**^a^** We adjusted for age, gender, comorbidity, nation, and year of THA surgery in overall analyses. In stratified analyses, we adjusted for all variables expect the stratification variable. For each stratification category a new Cox model was fitted.

The risk of revision within 90 days was 0.7% among cemented and 1.4% among cementless THA.

### Risk of 14- and 30-day mortality

Mortality within 14 days was also analyzed, and during this time frame 158 patients (0.14%, CI 0.10–0.19) with cemented THA and 72 (0.08%, CI 0.05–0.13) who had received cementless THA died, translating into an unadjusted HR of 1.6 (CI 1.2–2.1) when comparing cemented with cementless THA (Table 4). When we fitted a Cox regression model adjusting for age, sex, CCI score, nation, and year of index THA, an adjusted HR of 0.91 (CI 0.64–1.3) for cemented compared with cementless THA was attained (Table 4).

**Table 4.** Crude and adjusted hazard ratios (HR) with 95% confidence intervals (CI) for 0–14- and 0–30-day mortality comparing cemented with cementless total hip arthroplasties

Fixation	Number of deaths	Number at risk	Crude HR (95% CI)	Adjusted HR (95% CI)
0–14-day mortality				
Cementless	72	80,034	Reference	Reference
Cemented	158	108,572	1.6 (1.2–2.1)	0.91 (0.64–1.3)
0–30-day mortality				
Cementless	104	80,034	Reference	Reference
Cemented	234	108,572	1.7 (1.3–2.1)	0.94 (0.71–1.3)

HR adjusted for age, gender, year of surgery, and comorbidity.

Mortality within 30 days was 0.21% (CI 0.17–0.26) for cemented THA and 0.12% (CI 0.09–0.17) for cementless fixation. The adjusted HR was 0.94 (CI 0.71–1.3) for cemented compared with cementless THA within 30 days of THA.

There was no statistically significant interaction between age and fixation, nor between sex, nation, and year of surgery and fixation within either 14 or 30 days (all p values were > 0.05).

The Aalen additive risk model confirmed a minor cumulated increased excess risk of death after cemented compared with cementless THA in the initial period after THA (up to 10 days), whereas no difference in the risk was observed afterwards (Figure). Overall, the Aalen model suggested no effect of cementation on the mortality up to 90 days. Since the intensity is low (i.e., a low risk of dying) the curve can be interpreted as (almost) the cumulative additional number of dead patients for cemented (compared with cementless) over time.

## Discussion

### Main findings

In this hitherto largest register study on the risk of death after THA accounting for comorbidity as an important confounder, we find no clinically relevant difference in the absolute or relative risk of death up to 90 days for cemented compared with cementless fixation.

Cementing is the gold standard for implant fixation in elderly patients (Mäkelä et al. 2014a). Even though cemented fixation is considered superior in terms of implant survival within the 1st decade, the surgeons fear BCIS, with at worst cardiac arrest and death of the patient (Donaldson et al. 2009). Due to its scarcity, risk factors behind BCIS are difficult to investigate; however, anecdotal evidence and a previous investigation into early mortality after THA including classical and reverse hybrids indicates that cementation of the femoral but not the acetabular component is the most dangerous moment (Garland et al. 2017).

The trend towards using cementless THA may be driven by forceful marketing and production-oriented streamlining of operative procedures, as well as shorter operating times and focus on risk of BCIS (Troelsen et al. 2013, Dale et al. 2019). The true incidence of death secondary to BCIS is unknown in patients undergoing THA due to osteoarthritis, but BCIS seems to be more frequent in elderly patients receiving hemiarthroplasty due to femoral neck fracture compared with elective patients with osteoarthritis receiving THA (Olsen et al. 2014). Previous register studies indicate that the risk of BCIS and even delayed thromboembolic events may have been overestimated, at least in patients receiving THA for reasons other than femoral neck fracture. Comparisons of cemented with cementless fixation support the notion that cementation is associated with a very small mortality risk increase, if at all, both in absolute and in relative terms (Dale et al. 2019, Ekman et al. 2019).

As an exception to this, an analysis based on data from the National Joint Registry of England & Wales dataset, led the authors to conclude that there is a statistically significant 11% increased risk of death associated with cemented compared with cementless THA (McMinn et al. 2012), but this interpretation has been contested (Whitehouse et al. 2014). Likewise, we showed no difference overall and in the most representative age groups, namely 60–69, 70–79, and +80 years of age. Our observation of an increased relative risk of mortality for cemented versus cementless THA only in the youngest age group is unexpected, but it is most likely the result of a combination of residual confounding, selection bias, and chance.

### Strengths and weaknesses of this study

The important strength of our study is the unique collaboration of 4 national registers used to establish the NARA database, comprising a high number of patients with information on comorbidity. This is particularly important when studying rare outcomes such as early death after THA surgery. The quality of the participating national hip registries including both completeness and accuracy of data is high (Pedersen and Fernstad 2016). Adjustment for comorbidity in studies on mortality after THA surgery is important, but only a few studies on samples of similar size to ours have access to complex health administrative datasets containing information on inpatient diagnoses prior to surgery, and the use of arthroplasty registry data alone to model postoperative mortality has been criticized (Whitehouse et al. 2014). Thus, in some studies, the ASA classification is used as a proxy of comorbidity, but internal variability within this classification is high. The considerably more complex CCI as a measure of comorbidity is a well-established tool to adjust for comorbidity in studies aimed at investigating short-term mortality after THA (Pedersen et al. 2011, Garland et al. 2017). Any misclassification of CCI is most probably not related to the mode of THA fixation since the underlying healthcare data is prospectively and routinely collected.

Our study has many limitations. Our choice of 90-day mortality as outcome can be questioned although the incidence of adverse events within 90 days after surgery is regularly reported as a measure of complications by many established arthroplasty registers. The longer time has elapsed after major surgery the less likely it is that the fixation mode of the index THA is related to the event of death (Pedersen et al. 2011). THA patients are elderly and can die due to numerous other reasons such as comorbid conditions or complications unrelated to the THA fixation technique. Thus, some studies investigating mortality after THA surgery performed due to fracture or osteoarthritis focus on earlier time points, and some address each day after surgery by a separate logistic regression analysis (Dale et al. 2019, Ekman et al. 2019). We therefore additionally explored 14- and 30-day mortality as measures of death occurring earlier after index surgery, with findings supporting our main conclusions. In order to account for risk modifications early during the observation period we also undertook Aalen additive risk modelling and found that there was a small risk increase associated with cemented fixation during the first 14 days after surgery but not thereafter.

Socioeconomic factors are associated with morbidity, mortality, and patient-reported outcome after THA, but we had no access to such information. However, a recent investigation into the added benefit of including socioeconomic background variables in analyses of early mortality after THA indicates that the level of comorbidity may, to a certain extent, serve as a proxy measure of socioeconomic background (Weiss et al. 2019). In addition, the Scandinavian healthcare systems provide public, tax-financed care, making socioeconomic factors less pronounced. Further, we did not have information on lifestyle factors. It is possible that younger patients with cementless THA are more physically active and fitter, influencing their risk of suffering from both intraoperative and early postoperative complications. The NARA dataset contains no information on causes of deaths, which would have enabled us to explore whether the youngest age group dies for other reasons than elderly patients early after THA surgery. Heterogeneity in data from disparate sources and between-country variation can be problematic, which would affect our study design with data from 4 Nordic countries, but this seems primarily to be an issue when pooled data are used for individual survival-probability assessments, an approach not utilized in our study (Bartz-Johannessen et al. 2019). We also investigated revision rates early after index THA and found 0.7% differences to the disadvantage of cementless fixation within 90 days, mostly due to a pronounced increased risk of periprosthetic fractures when compared with cemented fixation. If one used cementless fixation in order to avoid cementation-related mortality one would have to take the increased risk of early revision and the mortality associated with such secondary procedures into account. Finally, we had no information on the use of mechanical or chemical thromboprophylaxis, which are associated with 90-day mortality after THA (Hunt et al. 2013, Pedersen et al. 2019). If use of thromboprophylaxis is related to fixation method we would be confronted with residual confounding in our study. We did not examine the association between hybrid THA and mortality.

### Clinical implications

Our study is based on a large population of THA patients from 4 Nordic countries and examines the association between THA fixation and mortality, granting generalizability of the study results. We interpret the findings from this and other studies as supportive of the continued use of cemented fixation of THA performed due to osteoarthritis although an observational study such as ours cannot entirely establish causal relationships between mode of fixation and mortality.

## Conclusion

After adjustment for comorbidity as an important confounder, we observed similar early mortality between the 2 fixation techniques, cemented and cementless.
